# EspO-mediated neutrophil accumulation drives lethality during *Citrobacter rodentium* infection in interleukin-22-deficient mice

**DOI:** 10.1371/journal.ppat.1014567

**Published:** 2026-09-02

**Authors:** Vishwas Mishra, Priyanka Biswas, Zuza Kozik, Jyoti Choudhary, Gad Frankel

**Affiliations:** 1 Department of Life Sciences, Imperial, London, United Kingdom; 2 Functional Proteomics Group, Chester Beatty Laboratories, Institute of Cancer Research, London, United Kingdom; University of Toronto, CANADA

## Abstract

*Citrobacter rodentium* (CR) is a murine-specific enteric pathogen widely used to model infections of the attaching and effacing pathogens enteropathogenic and enterohaemorrhagic *Escherichia coli*. Infection induces colonic epithelial damage and inflammation that are resolved in resistant mice but result in severe disease and mortality in mice lacking interleukin-22 (IL-22). While IL-22-deficient (*Il22*^*−/−*^) mice succumb to CR infection due to dehydration, in part mediated by disruption of the barrier integrity by the type III secretion system (T3SS) effector EspF, the contribution of immunopathology to infection outcomes is not known. Here, using CR we tested whether dysregulated neutrophil responses drive disease progression in *Il22*^*−/−*^ mice. We show that IL-22 deficiency is associated with progressive colonic pathology and increased neutrophil accumulation and activity in the colonic mucosa. Infection of mice with CR lacking the T3SS effector EspO, which is implicated in neutrophil recruitment, resulted in markedly reduced neutrophil accumulation and activity in *Il22*^*−/−*^ mice without altering bacterial burden. Notably, CRΔ*espO* infection led to reduced colonic inflammation and complete survival of *Il22*^*−/−*^ mice. Consistent with these findings, CXCR2 antagonist treated *Il22*^*−/−*^ mice displayed attenuated disease severity and delayed mortality. Together, we identified EspO as the second effector, alongside EspF, whose deletion results in survival of CR-infected *Il22*^*−/−*^ mice. Moreover, these findings suggest that excessive neutrophil accumulation and activity are key drivers of lethality during CR infection in *Il22*^*−/−*^ mice and that EspO amplifies neutrophil-dependent pathology within a vulnerable epithelial environment.

## Author summary

The immune system protects the intestine from infection, but excessive inflammation can itself cause severe tissue damage and disease. Interleukin-22 (IL-22) is a key cytokine that supports intestinal epithelial defence during infection with *Citrobacter rodentium* (CR), a bacterial pathogen commonly used to model human diarrhoeal disease. Mice lacking IL-22 develop severe lethal colitis following CR infection, but the role of immunopathology in this outcome has remained unclear. Here, we show that IL-22-deficient mice develop excessive accumulation and activation of neutrophils in the colon during CR infection. Rather than being protective, these exaggerated neutrophil responses drive lethal immunopathology. Using deletion of the bacterial virulence factor EspO as a pathogen-encoded means to attenuate neutrophil recruitment, we demonstrate that reducing colonic neutrophil accumulation is sufficient to prevent mortality. Our findings identify dysregulated neutrophil responses as key determinants of CR-induced lethality in IL-22-deficient mice and reveal how a bacterial effector can amplify harmful inflammation in a vulnerable host environment.

## Introduction

*Citrobacter rodentium* (CR) is a murine-specific enteric pathogen widely used to model infections caused by the attaching and effacing pathogens enteropathogenic and enterohaemorrhagic *Escherichia coli* [1]. CR adheres intimately to the distal colonic epithelium and triggers mucosal inflammation [2–4]. Disease severity is shaped by host immune responses and bacterial virulence determinants [5]. Central to CR pathogenesis is the locus of enterocyte effacement (LEE)-encoded type III secretion system (T3SS), which delivers effector proteins that modulate host cellular signalling [1,2]. Although several T3SS effectors are individually dispensable for colonisation, they function as a coordinated network with context-dependent roles in disease progression [5–7]. EspO, which binds integrin-linked kinase and Hax-1 [8,9], is an effector implicated in colonic colonisation, epithelial repair, and neutrophil recruitment [5,8,10–12], though the mechanism remains poorly defined.

Interleukin-22 (IL-22) is produced by innate and adaptive lymphocytes and acts on epithelial cells to maintain barrier integrity, antimicrobial defence, and tissue repair [13]. Accordingly, IL-22-deficient (*Il22*^*−/−*^) mice develop severe colitis and succumb to CR infection, independent of the gut microbiota [14,15]. We recently demonstrated that mortality in *Il22*^*−/−*^ mice results from systemic dehydration secondary to severe colonic epithelial damage and inflammation, rather than impaired epithelial repair or bacterial control [16]. However, the specific host factors and inflammatory pathways driving this pathology in the absence of IL-22 remain incompletely defined.

Neutrophils are rapidly recruited to the CR infection sites and are essential for bacterial containment and mucosal defence [17]. However, excessive activation has been linked to epithelial injury, colitis, and mortality in susceptible hosts [18]. In *Il22*^*−/−*^ mice, conflicting observations have been reported. Impaired neutrophil recruitment has been proposed as a driver of severe CR-induced disease in *Il22*^*−/−*^ mice [19], whereas recent studies demonstrated increased neutrophil accumulation [16,20]. Thus, how neutrophil responses are regulated in *Il22*^*−/−*^ mice and contribute to disease outcomes, remain unresolved.

Here, we investigate how IL-22 deficiency alters neutrophil responses during CR infection and whether excessive neutrophil accumulation and activity drive disease outcomes. Using CRΔ*espO* and a selective CXCR2 antagonist as complementary approaches to limit neutrophil accumulation, we investigated the contribution of neutrophils to lethal CR infection in *Il22*^*−/−*^ mice.

## Results

### CR infection in *II22*^*−/−*^ mice leads to neutrophil-associated epithelial injury

**Fig 1:**
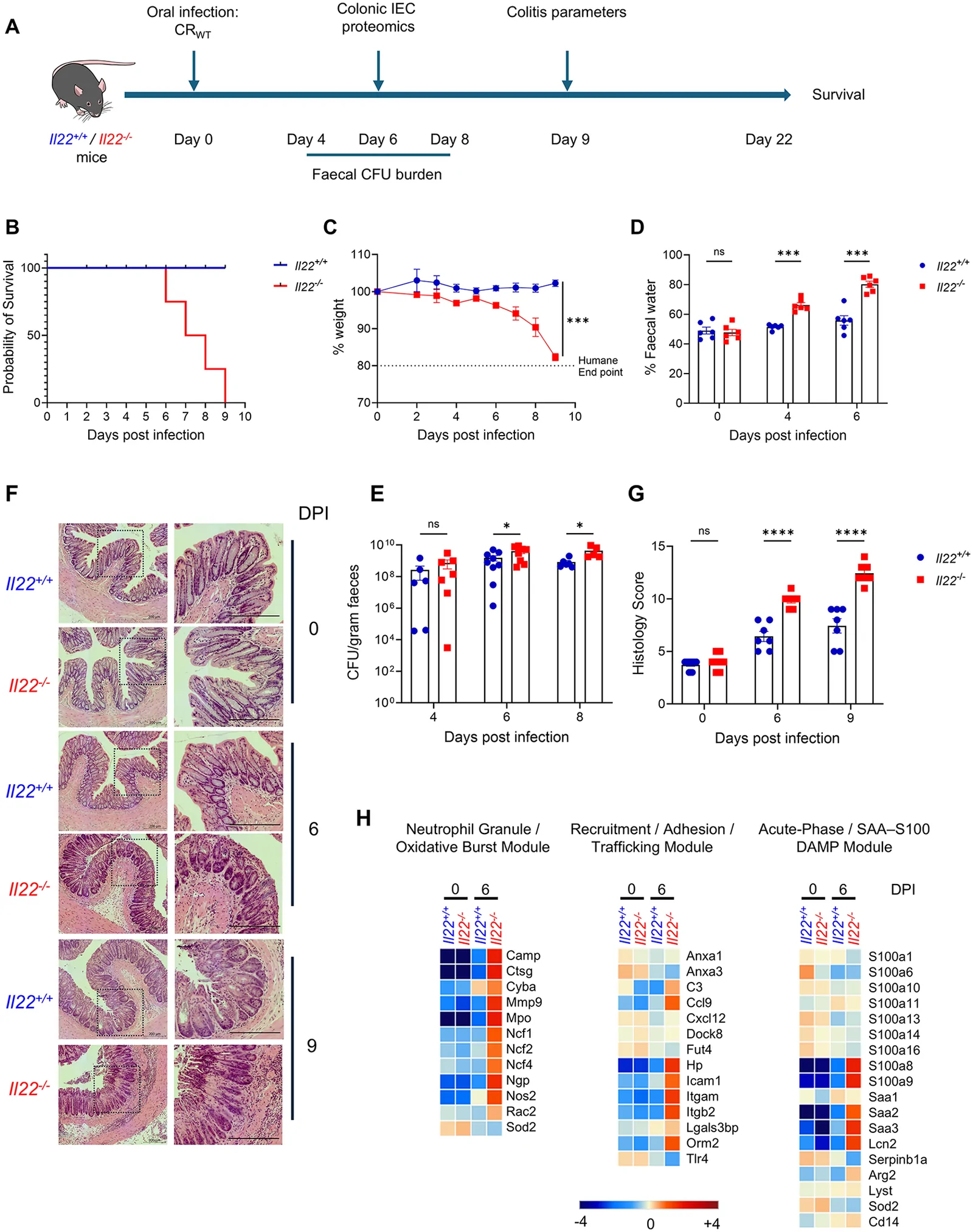
*Il22*^−/−^ mice display neutrophil-associated epithelial injury during CR infection. (**A)** Experimental timeline showing oral infection of *Il22*^*+/+*^ and *Il22*^*−/−*^ mice with CR. **(B)** Survival analysis. **(C)** Body weight change over the course of infection**. (D-E)** Faecal water content **(D)** and CR burden **(E)** at indicated dpi. **(F-G)** Representative H&E-stained distal colon sections **(F)** histological scores **(G)** from *Il22*^*⁺/⁺*^ and *Il22*^*−/−*^ mice at 0, 6, and 9 dpi. Scale 200µm. **(H)** Heatmaps of cIECs-enriched proteomic modules associated with neutrophil granule/oxidative burst pathways, leukocyte recruitment and adhesion, and acute-phase/DAMP responses, highlighting early induction of neutrophil-associated injury programs in *Il22*^*−/−*^ mice. Data shown in **(B-C)** are representative of 2 biological repeats, with 4 and 5 mice each per group, respectively. Each dot in **(D-E, G)** represents a mouse and are pooled values from 2 biological repeats, with 4-5 mice per group. P values were determined on data plotted as mean ± SEM using Two-way ANOVA with Bonferroni post-test for multiple comparisons. ns, non-significant; *, p < 0.05; ***, p < 0.001; ****, p < 0.0001. The mouse cartoon in **(A)** is an Illustration from NIAID NIH BioArt Source (bioart.niaid.nih.gov/bioart/281).

Consistent with previous reports [15,16], *Il22*^*−/−*^ mice developed severe disease following CR infection, exhibiting rapid weight loss, diarrhoea and mortality by 10 dpi (Fig 1A-D). Faecal bacterial burdens were comparable between genotypes at 4 dpi, with *Il22*^*−/−*^ mice displaying increased burdens at later time points (Fig 1E). Histological analysis of the distal colon at 6 and 9 dpi revealed markedly exacerbated pathology in *Il22*^*−/−*^ mice (Fig 1F-G), demonstrating progressive epithelial injury and inflammation during infection.

To study epithelial responses underlying this progression, we performed proteomic profiling of colonic epithelial-enriched fractions isolated from uninfected *Il22*^*+/+*^ and *Il22*^*−/−*^ mice and at 6 dpi. Principal component analysis demonstrated a distinct proteomic profile in epithelial-enriched fractions isolated from infected *Il22*^*−/−*^ mice compared with *Il22*^*+/+*^ controls (S1 Fig). Enriched pathways included neutrophil-associated epithelial injury, acute-phase and damage-associated molecular pattern responses (SAA-S100 proteins), oxidative stress and reactive oxygen species handling, and leukocyte recruitment, adhesion, and trafficking (Fig 1H). Importantly, neutrophil-associated proteins were also enriched, suggesting the presence of infiltrating neutrophils within the epithelial-enriched fractions.

Notably, these epithelial alterations were evident early during infection, preceding overt tissue collapse and mortality. Together, these findings indicate that IL-22 deficiency results in epithelial injury-prone state characterised by induction of neutrophil-associated damage and stress pathways.

### *Il22*^*−/−*^ mice exhibit exaggerated neutrophil accumulation and activity during CR infection

**Fig 2:**
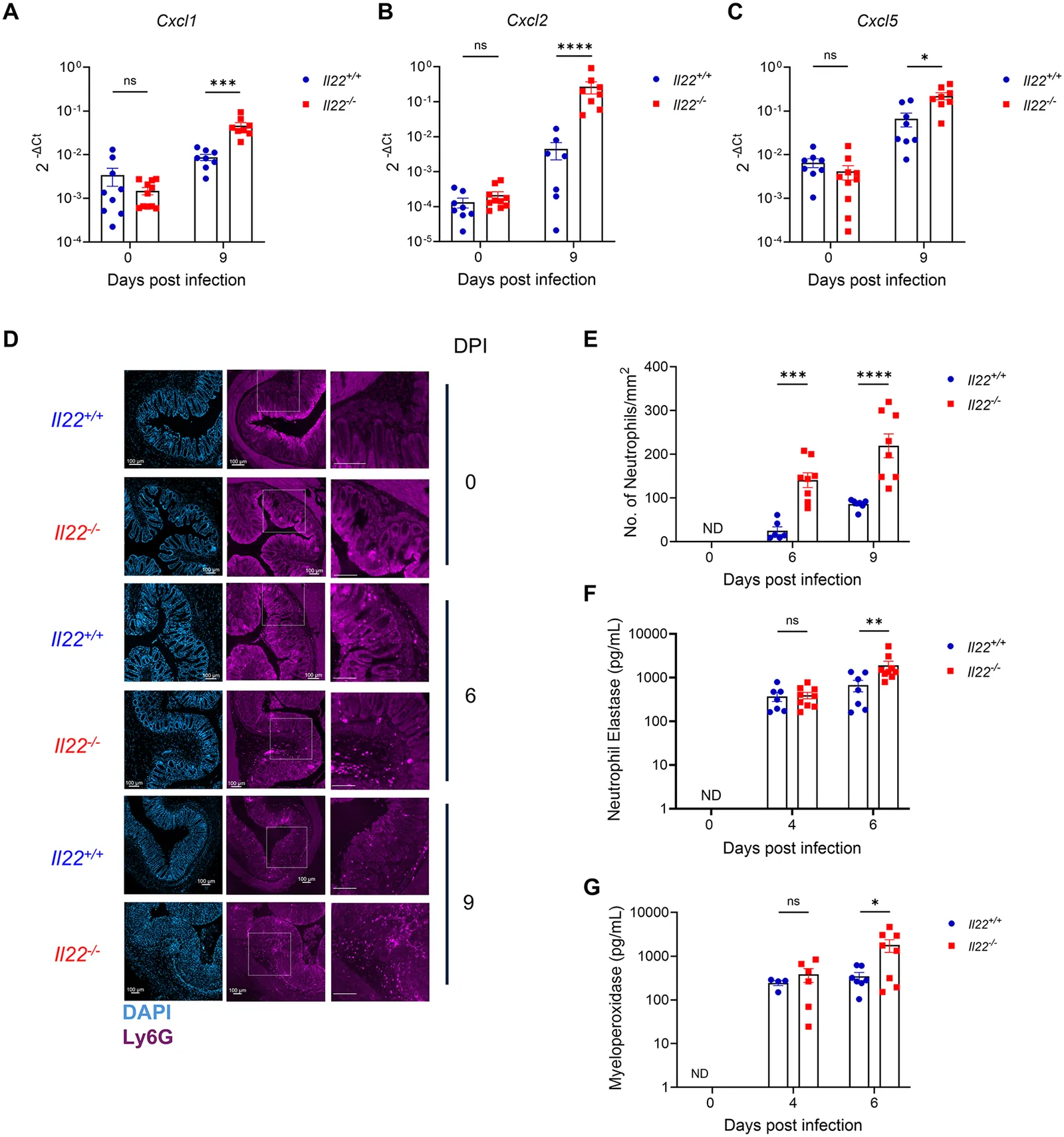
CR infected *Il22*^−/−^ mice display excessive colonic neutrophil accumulation. **(A-C)** qRT-PCR analysis of neutrophil-attractant chemokines *Cxcl1*, *Cxcl2*, and *Cxcl5* in whole colonic tissue from *Il22*^*+/+*^ and *Il22*^*−/−*^ mice at indicated dpi. **(D-E)** Representative immunofluorescence images of distal colon sections **(D)** and quantification of Ly6G⁺DAPI^+^E-cadherin^-^ neutrophils **(E)** at indicated dpi. Scale 100µm. Also see Fig. S2. **(F-G)** Faecal NE **(F)** and MPO **(G)** levels at indicated dpi. Each dot in (**A-C**, **E-G**) represents a mouse and are pooled values from 2 biological repeats, with 4-5 mice per group. P values were determined on data plotted as mean ± SEM using Two-way ANOVA with Bonferroni post-test for multiple comparisons. ns, non-significant; *, p < 0.05; **, p < 0.01; ***, p < 0.001; ****, p < 0.0001.

Given enrichment of neutrophil-associated proteins and injury pathways in epithelial-enriched proteomic analysis, we assessed neutrophil accumulation and activity in the distal colon. qRT-PCR analysis showed comparable basal expression of *Cxcl1*, *Cxcl2*, and *Cxcl5* in uninfected *Il22*^*+/+*^ and *Il22*^*−/−*^ mice (Fig 2A-C), however, CR-infected *Il22*^*−/−*^ mice exhibited significantly higher expression than *Il22*^*+/+*^ controls (Fig 2A-C). Consistent with this, immunofluorescence demonstrated increased Ly6G⁺E-cadherin^-^ neutrophil accumulation in the colonic mucosa of *Il22*^*−/−*^ mice, which progressed during infection and coincided with severe epithelial pathology (Fig 2D-E, S2 Fig).

To assess neutrophil activity, we quantified faecal neutrophil elastase (NE) and myeloperoxidase (MPO). While levels were comparable at 0 and 4 dpi, *Il22*^*−/−*^ mice displayed significantly elevated NE and MPO at 6 dpi relative to controls (Fig 2F-G).

To further characterise the accumulating neutrophils, we reanalysed our previously published flow cytometry dataset from the colonic lamina propria of CR-infected mice using a refined gating strategy focused on innate immune populations (S7 Fig) [16]. Consistent with our original analysis [16], no differences were observed in monocyte or macrophage populations between genotypes (S3A- S3B Figs). In contrast, *Il22*^*−/−*^ mice exhibited significantly increased Ly6G^+^CD11b^high^ mature neutrophils, whereas Ly6G^+^CD11b^low^ immature neutrophils were comparable between groups (S3C-S3D Figs).

Together, these data indicate that IL-22 deficiency promotes the accumulation and activation of neutrophils during CR infection.

### Deletion of *espO* attenuates neutrophil accumulation and prevents lethal CR infection in *Il22*^*−/−*^ mice

**Fig 3:**
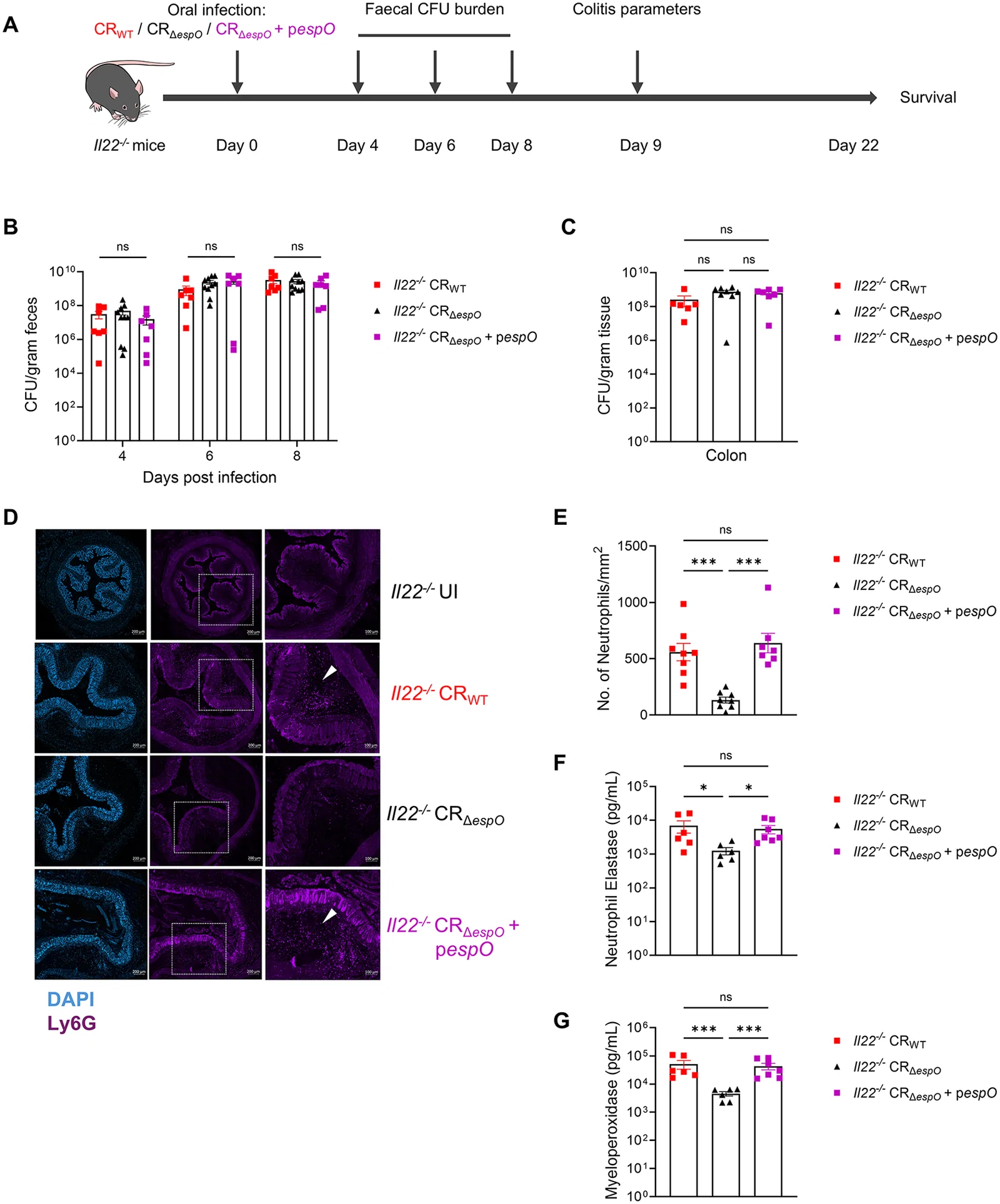
CRΔespO causes lower neutrophil accumulation and activity in *Il22*^−/−^ mice. **(A)** Experimental timeline showing oral infection of *Il22^−/−^* mice with CRWT, ΔespO or ΔespO  +  pespO strains. **(B-C)** Faecal bacterial burden at indicated dpi **(B)** and colonic bacterial burden at 9 dpi **(C)**. **(D-E)** Representative immunofluorescence images of distal colon sections **(D)** and quantification of Ly6G⁺DAPI+E-cadherin- neutrophils **(E)** at indicated dpi. Scale 200 µm. **(F-G)** Faecal NE **(F)** and MPO **(G)** levels demonstrating reduced neutrophil effector activity in CRΔespO-infected mice. Each dot in (**B-C**, **E-G**) represents a mouse and are pooled values from 2 biological repeats, with 3-5 mice per group. P values were determined on data plotted as mean  ±  SEM using Two-way ANOVA **(B)** and One-way ANOVA **(C, E-G)** with Bonferroni post-test for multiple comparisons. ns, non-significant; *, p  <  0.05; ***, p  <  0.001. The mouse cartoon in **(A)** is an Illustration from NIAID NIH BioArt Source (bioart.niaid.nih.gov/bioart/281).

To determine whether exaggerated neutrophil responses drive lethal disease in *Il22*^*−/−*^ mice, we exploited a deletion mutant lacking the CR T3SS effector EspO, which promotes neutrophil recruitment during infection [10,11], as a pathogen-encoded means to attenuate neutrophil responses. *Il22*^*−/−*^ mice were infected with wildtype CR (CR_WT_), an isogenic Δ*espO* mutant, or the complemented strain (Δ*espO* + p*espO*) [10]) (Fig 3A). Faecal and colonic-associated bacterial burdens were comparable between groups, indicating that EspO is dispensable for intestinal colonisation of *Il22*^*−/−*^ mice (Fig 3B-C).

CRΔ*espO* infection markedly reduced Ly6G^+^E-cadherin^-^ neutrophil accumulation within the distal colonic mucosa together with lower faecal NE and MPO levels, indicating reduced neutrophil recruitment and activation (Fig 3D-G). Complementation with p*espO* restored these responses to CR_WT_ levels.

**Fig 4:**
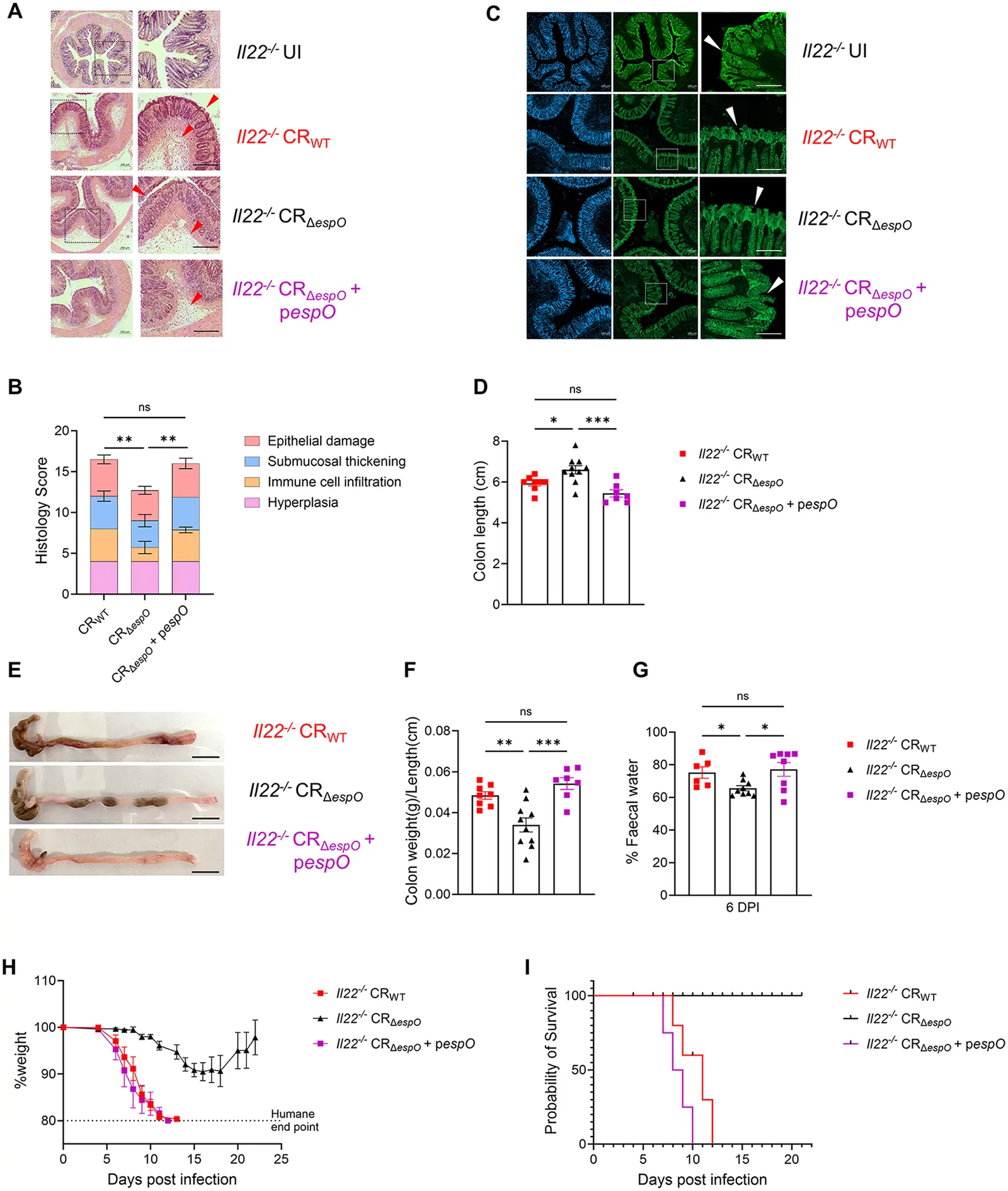
Attenuated neutrophil accumulation results in survival of *Il22*^−/−^ mice. **(A-B)** Representative H&E-stained distal colon sections **(A)** and histological scoring based on epithelial damage, submucosal thickening, immune cell infiltration and hyperplasia **(B)** at 9 dpi. Scale 200 µm. Note the similar submucosal thickening with lower immune cell infiltration in CRΔespO-infected compared to CR WT infected mice. Also see Fig. S4 and Fig. S5A. **(C)** Representative immunofluorescence images of distal colon sections stained for E-cadherin. Scale 100 µm. Note the epithelial damage and abrasion. **(D-G)** Colon length **(D)**, representative images of colons **(E)**, colon weight-to-length ratios **(F)** and faecal water content **(G)** from *Il22^−/−^* mice infected with CR WT, CRΔespO or CRΔespO  +  pespO strains at 9 dpi. **(H-I)** Body weight change over the course of infection **(H)** and survival curves **(I)** in infected *Il22^−/−^* mice. Each dot in **(B, D, F-G)** represents a mouse and are pooled values from 2 biological repeats, with 3-5 mice per group. Data shown in **(H-I)** are representative of 2 biological repeats, with 3-5 mice each per group. P values were determined on data plotted as mean  ±  SEM using One-way ANOVA with Bonferroni post-test for multiple comparisons. P values for **(B)** were determined on total histological scores, see Fig. S5A. ns, non-significant; *, p  <  0.05; **, p  <  0.01; ***, p  <  0.001.

Despite reduced neutrophil accumulation, CRΔ*espO*-infected *Il22*^*−/−*^ mice exhibited epithelial injury comparable to CR_WT_, including similar submucosal thickening, epithelial damage and E-cadherin disruption (Fig 4A-C, S4 Fig, S5A Fig). Consistent with this, bacterial dissemination to the liver and spleen was similar between groups (S5B-C Fig). In contrast, inflammatory cell infiltration was markedly reduced in CRΔ*espO*-infected mice, which also displayed increased colon length, lower colon weight-to-length ratios and lower faecal water content, indicating reduced colitis (Fig 4A-B, 4D-G). Importantly, CRΔ*espO* infection resulted in 100% survival with significantly attenuated weight loss compared with CR_WT_ (Fig 4H-I). These phenotypes were restored to CR_WT_ levels by p*espO* complementation.

To further validate the contribution of neutrophil accumulation and activity to disease, CR_WT_ infected *Il22*^*−/−*^ mice were treated with the CXCR2 antagonist, SB225002 [21,22]. SB225002 treatment resulted in reduced weight loss and faecal water content without affecting bacterial shedding and resulted in delayed predicted mortality (S6 Fig).

Together, these findings identify excessive neutrophil accumulation as a key determinant of lethal disease during CR infection in the absence of IL-22.

## Discussion

This study demonstrates that neutrophil accumulation and activity play a key role in CR-induced disease progression and lethality in *Il22*^*−/−*^ mice. By using a strain lacking the T3SS effector EspO and CXCR2 antagonist to attenuate neutrophil responses, we show that excessive neutrophil accumulation and activation underlie fatal disease. These findings refine current models of enteric infection in *Il22*^*−/−*^ mice by identifying neutrophil-dependent pathology as a critical outcome determinant. Notably, EspO represents only the second CR effector, alongside EspF [23], whose deletion results in survival of *Il22*^*−/−*^ mice during infection.

The inflammatory mechanisms responsible for CR-mediated severe colonic inflammation in *Il22*^*−/−*^ mice have remained unclear. IL-22-STAT3 signalling promotes epithelial expression of neutrophil-attractant chemokines [24]. Therefore, it was previously proposed that reduced neutrophil recruitment may contribute to disease severity in the absence of IL-22 [19]. In contrast, recent studies showed higher neutrophil accumulation in *Il22*^*−/−*^ mice upon CR infection [16,20]. Our findings demonstrate elevated expression of neutrophil-attracting chemokines (*Cxcl1*, *Cxcl2*, and *Cxcl5*) in whole colonic tissue and suggest exaggerated neutrophil accumulation and activity as key drivers of mortality in CR-infected *Il22*^*−/−*^ mice.

A transposon-based screen of ~1,800 Tn5-generated mutants identified the T3SS effector EspF as a critical mediator of tight junction disruption and fatal inflammation in CR-infected *Il22*^*−/−*^ mice [23]. EspO was not identified, likely due to its small coding sequence, as it is the smallest CR effector (10 kDa). Consistent with this, *Il22*^*−/−*^ mice infected with CRΔ*espO*, which retain EspF, still developed epithelial injury, submucosal thickening, and weight loss. However, despite comparable colonic colonisation and persistent epithelial damage, *espO* deletion markedly reduced neutrophil accumulation and resulted in survival. Together, these findings indicate that while epithelial injury is necessary, deleterious neutrophil responses act as a critical downstream determinant of fatal disease in IL-22-deficient hosts.

EspO has orthologues in enteric bacterial pathogens, including *Shigella* (OspE), EPEC, EHEC and *Salmonella* [9]. Previous studies have implicated EspO in regulating neutrophil recruitment during CR infection, with Δ*espO* strains exhibiting reduced expression of neutrophil-attracting chemokines and Mmp9, accompanied by diminished submucosal neutrophil infiltration [8,10]. Our findings extend these observations by demonstrating that EspO plays an important role in neutrophil accumulation and lethality in *Il22*^*−/−*^ mice. However, further studies will be required to define the molecular mechanisms by which EspO modulates neutrophil recruitment within the inflamed intestinal mucosa, and how these processes intersect with epithelial injury and repair pathways.

More broadly, our findings illustrate how protective inflammatory responses become pathogenic when epithelial resilience is compromised. In the absence of IL-22, excessive neutrophil accumulation drives lethal disease despite comparable bacterial burden, highlighting inflammatory amplification rather than pathogen burden as a key determinant of outcome during severe enteric infection.

## Materials and methods

Detailed materials and methods, and raw data are available on Figshare documents entitled “Resources” and “Raw_Data”, respectively.

(DOI: https://doi.org/10.6084/m9.figshare.32960693)

### Mice and ethical approval

All animal experiments were approved by the Imperial College Animal Welfare and Ethical Review Body and conducted under UK Home Office regulations (PPL PP8896560) in accordance with the Animals (Scientific Procedures) Act 1986. Mice were housed under specific pathogen-free conditions (12 h light/dark cycle, 22 ± 2 °C) in individually ventilated cages with ad libitum access to RM1(E) diet (SDS Diets) and water. Male and female mice aged 2–3 months were used.

### CR infection and colitis analysis

CR strains were cultured, prepared, and administered by oral gavage (∼1 × 10⁹ CFU in 200 µL PBS) as previously described [11,25]. At experimental endpoints, mice were euthanised and the colon processed for macroscopic analysis, histology, RNA extraction and epithelial cell isolation [16].

For SB225002 treatment, CR-infected *Il22*^*−/−*^ mice received twice-daily intraperitoneal injections of SB225002 (1 mg/kg) in PBS containing 0.33% Tween-80 from 3 dpi; control mice received vehicle alone.

Paraffin-embedded colons were processed for histology and immunofluorescence as described previously [25]. Ly6G⁺E-cadherin^–^DAPI⁺ neutrophils were quantified using established image acquisition and analysis workflows [16].

### Proteomics analysis from colonic epithelial cells

Colonic epithelial-enriched fractions were isolated, processed for protein extraction, digestion and TMT labelling, and analysed by mass spectrometry as previously described [16]. Proteomic data were processed as described previously [16]. The dataset has been deposited with the ProteomeXchange Consortium via PRIDE under accession PXD074250.

### Statistical analysis

Experiments were performed in randomised blocks (3–5 mice per genotype) and repeated independently at least twice. Data were pooled and are presented as mean ± SEM. Statistical analyses were performed using GraphPad Prism (v10.4.0); details are provided in the corresponding figure legends.

## Supporting information

S1 Fig Proteomic differences between *Il22*^*+/+*^ and *Il22*^*−/−*^ mice in epithelial-enriched fractions during CR infection. **(A)** Principal component analysis (PCA) of proteomic profiles from epithelial-enriched colonic fractions isolated from *Il22*^*+/+*^ and *Il22*^*−/−*^ mice infected with CR at 6 dpi. **(B)** Volcano plot showing differential protein abundance between *Il22*^*−/−*^ and *Il22*^*+/+*^ at 6 dpi. Proteins significantly increased (red), decreased (blue), or unchanged (grey) in *Il22*^*−/−*^ mice are indicated.
(TIF)

S2 Fig Increased neutrophil accumulation during CR infection in *Il22*^*−/−*^ mice. Immunofluorescence staining for Ly6G in distal colonic sections from *Il22*^*+/+*^ and *Il22*^*−/−*^. The images show staining for the complete colon section for representative images shown in Fig. 2D. Scale 200µm.
(TIF)

S3 Fig CR infected *Il22*^*−/−*^ mice display higher mature neutrophils in the colon. Reanalysis of flow cytometry data from lamina propria of CR infected *Il22*^*+/+*^ and *Il22*^*−/−*^ mice at 9 dpi previously published [16] using refined gating strategy; see Fig. S7. Absolute numbers of **(A)** Ly6C^+^F480^-^ Monocytes, **(B)** F480^+^Ly6C^-^ Macrophages, **(C)** Ly6G^+^Cd11b^high^ mature neutrophils and **(D)** Ly6G^+^Cd11b^low^ immature neutrophils. Each dot represents a mouse. P values were determined on data plotted as mean ± SEM using Two-way ANOVA with Bonferroni post-test for multiple comparisons. ns, non-significant; *, p < 0.05.
(TIF)

S4 Fig CRΔ*espO* infected *Il22*^*−/−*^ mice display lower immune cell infiltration. Representative H&E-stained distal colon sections from *Il22*^*−/−*^ mice (n = 3 per group) infected with either CR WT, Δ*espO* or Δ*espO* + p*espO* showing comparable epithelial damage and submucosal thickening, with reduced immune cell infiltration in Δ*espO*-infected mice. Scale 200µm.
(TIF)

S5 Fig CRΔ*espO* infected *Il22*^*−/−*^ mice display similar extra intestinal CR burden. **(A)** Total histological scores based on epithelial damage, submucosal thickening, immune cell infiltration and hyperplasia. **(B-C)** Liver CFU **(B)** and spleen CFU **(C)** from infected *Il22*^*−/−*^ mice at 9 dpi. Each dot represents a mouse and are pooled values from 2 biological repeats, with 3–5 mice per group. P values were determined on data plotted as mean ± SEM using One-way ANOVA with Bonferroni post-test for multiple comparisons. ns, non-significant; **, p < 0.01.
(TIF)

S6 Fig CXCR2 antagonist treatment results in delayed mortality in *Il22*^*−/−*^ mice. (**A)** Schematic showing the experimental strategy. *Il22*^*−/−*^ mice were orally infected with CR WT, 3 dpi onwards mice were either treated with 1 mg/kg body weight of SB225002 or vehicle (VC). Mice were monitored daily for disease signs and symptoms. Mice were euthanised when they reach humane end point. **(B-C)** Faecal CFU **(B)** and water content **(C)** at 6 dpi. **(D)** Weight loss upon infection. **(E)** Survival analysis post infection. Each dot in **(B-C)** represents a mouse and are pooled values from 2 biological repeats, with 4–5 mice per group. Data shown in **(D-E)** are pooled of 2 biological repeats, with 4–5 mice each per group. P values were determined on data plotted as mean ± SEM using Student’s t-test **(B-C)**. For **(D)** area under the curve was calculated with SD and Student’s t-test was used to determine the p value. P value for survival in **(E)** was calculated using Mantel-Cox test. ns, non-significant; *, p < 0.05; **, p < 0.01; ***, p < 0.001. The mouse cartoon in **(A)** is an Illustration from NIAID NIH BioArt Source (bioart.niaid.nih.gov/bioart/281).
(TIF)

S7 Fig Gating strategy for reanalysis of FACS data. The raw fcs files corresponding to data already published [16] were reanalysed using the refined gating strategy to determine the number of Ly6G^+^ Cd11b^high^ mature and Ly6G^+^ Cd11b^low^ immature neutrophils in the colonic lamina propria at 9 dpi. Also see Fig. S3.
(TIF)
