# Supplementary material for: EspO-mediated neutrophil accumulation drives lethality during Citrobacter rodentium infection in interleukin-22-deficient mice
Source: PLoS Pathog. 2026 Sep 2;22(9):e1014567. doi: 10.1371/journal.ppat.1014567 (PMC13577462; doi:10.1371/journal.ppat.1014567)
Supplement: Supplementary file 1 [file ppat.1014567.s008.pdf]

Response to reviewers (PPATHOGENS-D-26-00394)

We thank the reviewer for their thoughtful and constructive comments, which have helped improve the rigour, clarity, and overall quality of our manuscript.

In response to the reviewer's suggestions, we have performed additional experiments and analyses. These include validation of our key findings using the complemented ΔespO + p*espO* strain and an independent in vivo pharmacological study using the CXCR2 inhibitor SB225002 to directly assess the contribution of neutrophil recruitment to mortality in *Il22*$^{-/-}$ mice. We have also reanalysed our flow cytometry data, included additional supplementary data, and revised the text throughout the manuscript to address the reviewer's concerns and improve clarity.

Our point-by-point responses to each comment are provided below.

Reviewer # 1

Comment 1. Inclusion of an EspO chromosomally complemented CR strain in the background of ΔespO CR would provide important validation of the key findings presented in Figures 3 and 4.

Response: We thank the reviewer for this critical suggestion. We have repeated the experiments presented in **Figures 3** and **4** using the *Citrobacter rodentium* (CR) Δ*espO* strain complemented with a plasmid expressing espO (Δ*espO* + p*espO*). This complemented strain was previously generated and validated in our earlier study, where it restored all phenotypes associated with deletion of *espO* (Berger *et al.*, *PLOS Pathogens*, 2018; PMID: 30365535).

To verify plasmid stability during infection, faecal samples from mice infected with the complemented strain were plated on both kanamycin (Selects for CR_{WT}) and kanamycin plus chloramphenicol (Selects for presence of p*espO*) agar. Comparable bacterial counts on both selective media confirmed that the plasmid was retained throughout the course of infection.

Importantly, complementation restored the phenotype observed with the wild-type strain. Infection with the Δ*espO* + p*espO* strain resulted in mortality of *Il22*$^{-/-}$ mice and recapitulated the CR wildtype-infected phenotype across all parameters examined, confirming that the attenuated phenotype of the Δ*espO* mutant was specifically attributable to loss of *espO*. These new data have been incorporated into the revised **Figures 3** and **4**.

Reviewer #2

Comment 1. The CR infection also appears to be associated with accumulation of other immune cells, including monocytes/macrophages, dendritic cells, and T cells. Did the authors

assess differences in these populations between *Il22*$^{-/-}$ and *Il22*$^{+/+}$ mice? Analysis of these populations could provide additional insight. Could the authors clarify the rationale for focusing primarily on neutrophils, and whether the potential contributions of other immune cells were considered?

Response: We thank the reviewer for this comment and agree that CR infection is associated with the accumulation of multiple immune cell populations. In our previous study, we demonstrated that infection induced infiltration of monocytes, macrophages and T cells, did not differ significantly between *Il22*$^{-/-}$ and *Il22*$^{+/+}$ mice. However, neutrophils showed significantly higher accumulation in the colon of *Il22*$^{-/-}$ mice compared to *Il22*$^{+/+}$ mice (Mishra *et al., Nature Communications,* 2025; PMID: 41361166).

In response to the reviewer's suggestion, and in conjunction with Comment 3, we have reanalysed the flow cytometry data from our previous study (Mishra *et al., Nature Communications,* 2025; PMID: 41361166) using a refined gating strategy (**New Fig. S7**), thereby maximising the information obtained from existing samples in accordance with the principles of the 3Rs (Replacement, Reduction and Refinement). Consistent with our original analysis, we found no significant differences in the abundance of monocytes or macrophages between *Il22*$^{-/-}$ and *Il22*$^{+/+}$ mice (**New Fig. S3A-B**). In contrast, *Il22*$^{-/-}$ mice exhibited a significant increase in the Ly6G^{+} CD11b^{high} population, corresponding to mature neutrophils, whereas the Ly6G^{+} CD11b^{low} population, representing immature neutrophils, was comparable between the two genotypes (**New Fig. S3B-C**).

These findings further support our rationale for focusing on neutrophils, as they represent the only immune cell population in our analysis that consistently differed between *Il22*$^{-/-}$ and *Il22*$^{+/+}$ mice and therefore constitute the most likely cellular contributor to the increased disease severity observed in the absence of IL-22.

Comment 2. Previous work from the group suggests that CRΔ*espO* reduces the expression of neutrophil-recruiting factors and neutrophil infiltration. In this study, Il22^{-}/$^{-}$ mice infected with CRΔespO show improved survival, despite no apparent change in epithelial damage. To better support a role for neutrophils in lethality, neutrophil depletion at early and later stages of CRwt infection could be informative.

Response: We agree that directly testing the contribution of neutrophils would further strengthen the study. To address this point, we performed pharmacological experiment using the selective CXCR2 antagonist SB225002, which has previously been shown to reduce neutrophil recruitment to the colon and improve survival in experimental *Clostridioides difficile* infection (Dong *et al., Gut Microbes,* 2023; PMID: 36951545).

Il22$^{-/-}$ mice infected with wild-type CR were treated with SB225002 (1 mg/kg body weight, twice daily) beginning at 3 days post-infection. SB225002 treatment significantly delayed mortality, producing a rightward shift in the survival curve, reduced body weight loss kinetics, and decreased faecal water content at 6 dpi (**New Fig. S6**), consistent with reduced colonic pathology and diarrhoea. Although CXCR2 inhibition did not completely prevent mortality, these findings provide independent evidence that excessive neutrophil recruitment is a major contributor to the lethal phenotype observed in *Il22*$^{-/-}$ mice following CR infection.

We did not perform neutrophil depletion at separate early and late stages of infection because disease progression in *Il22*$^{-/-}$ mice is exceptionally rapid. Mice typically become ill by 7 dpi and frequently reach humane endpoints at this stage, making it technically challenging to distinguish discrete early and late phases of disease. Nevertheless, the pharmacological inhibition experiment directly addresses the reviewer's central point by demonstrating that limiting neutrophil recruitment ameliorates disease severity and delays mortality.

Comment 3. Is this phenomenon independent of neutrophil development and maturation?

Response: While our data do not directly address whether the lethal phenotype is dependent on neutrophil development or maturation, our reanalysis of the flow cytometry data provides relevant insight. Specifically, *Il22*$^{-/-}$ mice exhibited a significant increase in the Ly6G^{+}CD11b^{high} population, corresponding to mature neutrophils, whereas the Ly6G^{+}CD11b^{low} population, representing immature neutrophils, was unchanged (**New Fig. S3**). These findings indicate that the phenotype is associated with increased accumulation of mature neutrophils within the colon rather than altered numbers of immature neutrophils. Please also see our response to Comment 1.

Comment 4. Can the bacteria still disseminate to other tissues?

Response: Yes, CR Δ*espO* was still able to disseminate to extra-intestinal tissues. Quantification of bacterial burden demonstrated comparable dissemination to the liver and spleen in mice infected with either wild-type CR or the Δ*espO* mutant (**New Fig. S5B-C**). These findings are consistent with our observation that epithelial damage, assessed by E-cadherin immunohistochemistry (**Fig. 4C**), was comparable between the two infection groups. Together, these data indicate that deletion of *espO* does not impair bacterial dissemination despite its marked effect on neutrophil accumulation and host survival.

Reviewer #3:

Comment 1. The authors perform proteomics of colonic epithelial cells at day 6 post-infection. However, as shown in fig. 2, at this timepoint there is a significant recruitment of neutrophils, and it is thus possible that the "distinct epithelial proteomic signature in IL-22ko mice" actually

includes neutrophil proteins. This is evident, for example, in Fig. 1G, where the "neutrophil granule/oxidative burst module" includes neutrophil proteins; for example, MPO is not an epithelial signature. Similarly, some of the acute phase/ S100 proteins are likely from neutrophils. I thus recommend that the authors rewrite this portion of the manuscript addressing the fact that the epithelial proteome likely includes neutrophil proteins; if not, additional controls to show the lack of neutrophil protein contamination need to be performed.

Response: We thank the reviewer and agree that the epithelial-enriched fractions are likely to contain a small proportion of infiltrating immune cells, particularly neutrophils, at this stage of infection. For this reason, we intentionally described these samples as epithelial-enriched fractions throughout the original manuscript rather than stating that they represented a pure epithelial cell population (**Line 85**).

In response to the reviewer's suggestion, we have revised the manuscript to further clarify this point. Specifically, we now explicitly acknowledge that proteins such as MPO, S100 family members, and other neutrophil-associated proteins detected in the epithelial-enriched fractions are likely to reflect the presence of infiltrating neutrophils rather than epithelial cell-intrinsic expression. Accordingly, these findings are interpreted as evidence of increased neutrophil association with the epithelial compartment rather than as epithelial-specific protein expression (**Lines 86-87, 91-92, 98**).

These revisions clarify the nature of the samples analysed while preserving the principal conclusions of the study.

Part III – Minor Issues: Editorial and Data Presentation Modifications

Reviewer #1:

Comment 1. The title could be refined to highlight “EspO-dependent dysregulation of neutrophil responses,” which would better reflect the central finding of the study.

Response: We thank the reviewer and we have now changed the title to “EspO-mediated neutrophil accumulation drives lethality during *Citrobacter rodentium* infection in interleukin-22-deficient mice”.

Comment 2. In the second paragraph of the Introduction (lines 56–62), it would be helpful to acknowledge the reported gut microbiota–independent lethality of CR infection in germ-free Il22⁻/⁻ mice (PMID: 39630719), to further contextualize the work.

Response: Thank you. This has been now done (**Line 59**).

Reviewer #2:

Comment 1. The methods should be described in more detail rather than simply referring to "as previously described."

Response: We thank the reviewer for this helpful suggestion. As this manuscript is submitted as a *Short Report*, the main text is subject to a strict word limit, which limits the level of methodological detail that can be included. In response to the reviewer's comment, we have now deposited the complete Methods and Resources have on Figshare to ensure that all protocols are fully accessible and to facilitate reproducibility. The public link has been mentioned under the Material and methods section (**Line 186-187**).

Private link: https://figshare.com/s/5a161b41837ff3691d60

Comment 2. Please correct the typos in Line # 84 ("of uninfected $Il22^{+/+}$ and $Il22^{-/-}$ mice") and Line # 288 ("with 4 and 5 mice each per group").

Response: This has now been done.

Comment 3. Could the authors provide higher-magnification overlays for Figures 2D, 3D, and 4B?

Response: We thank the reviewer for this suggestion. The figures already include higher-magnification images alongside the lower-magnification overview panels to highlight the relevant histological features. We considered providing images at an even higher magnification; however, doing so would substantially reduce the field of view and obscure the overall tissue architecture, thereby limiting interpretation of the findings. We therefore believe that the current combination of low- and high-magnification images provides the most informative presentation of the histological data.

Comment 4. Figure 4 would benefit from inclusion of histological scoring.

Response: This has now been done. (**New Fig. 4B, S5A**).
