# Supplementary material for: EspO-mediated neutrophil accumulation drives lethality during Citrobacter rodentium infection in interleukin-22-deficient mice
Source: PLoS Pathog. 2026 Sep 2;22(9):e1014567. doi: 10.1371/journal.ppat.1014567 (PMC13577462; doi:10.1371/journal.ppat.1014567)
Supplement: Peer review history

# Peer review: Decision Letter 0

**DOI:** 10.1371/journal.ppat.1014567.r001

**Authors:** Matthew C. Wolfgang, William Navarre

4 May 2026

PPATHOGENS-D-26-00394

Dysregulated neutrophil response drives lethality during Citrobacter rodentium infection in interleukin-22-deficient mice

PLOS Pathogens

Dear Dr. Mishra,

Thank you for submitting your manuscript to PLOS Pathogens. After careful consideration, we feel that it has merit but does not fully meet PLOS Pathogens's publication criteria as it currently stands. Therefore, we invite you to submit a revised version of the manuscript that addresses the points raised during the review process.

Please submit your revised manuscript by Jul 03 2026 11:59PM. If you will need more time than this to complete your revisions, please reply to this message or contact the journal office at plospathogens@plos.org. When you're ready to submit your revision, log on to https://www.editorialmanager.com/ppathogens/ and select the 'Submissions Needing Revision' folder to locate your manuscript file.

Please include the following items when submitting your revised manuscript:

* A letter that responds to each point raised by the editor and reviewer(s). You should upload this letter as a separate file labeled 'Response to Reviewers'. This file does not need to include responses to any formatting updates and technical items listed in the 'Journal Requirements' section below.

* A marked-up copy of your manuscript that highlights changes made to the original version. You should upload this as a separate file labeled 'Revised Manuscript with Track Changes'.

* An unmarked version of your revised paper without tracked changes. You should upload this as a separate file labeled 'Manuscript'.

If you would like to make changes to your financial disclosure, competing interests statement, or data availability statement, please make these updates within the submission form at the time of resubmission. Guidelines for resubmitting your figure files are available below the reviewer comments at the end of this letter.

We look forward to receiving your revised manuscript.

Kind regards,

William Navarre

Academic Editor

PLOS Pathogens

Matthew Wolfgang

Section Editor

PLOS Pathogens

Sumita Bhaduri-McIntosh

Editor-in-Chief

PLOS Pathogens

orcid.org/0000-0003-2946-9497

Michael Malim

Editor-in-Chief

PLOS Pathogens

orcid.org/0000-0002-7699-2064

**Additional Editor Comments:**

The manuscript was reviewed by three experts in the field, all of whom felt the work was significant and generally well controlled. A few points of concern were raised and the academic editor agrees that addressing these comments would greatly improve the work.

**Journal Requirements:**

1) <carina-action-element class="ng-star-inserted">Please upload all main figures as separate Figure files in .tif or .eps format. For more information about how to convert and format your figure files please see our guidelines: </carina-action-element>

2) We have noticed that you have uploaded Supporting Information files, but you have not included a list of legends. Please add a full list of legends for your Supporting Information files after the references list.

3) <carina-action-element class="ng-star-inserted">Please </carina-action-element><carina-action-element class="ng-star-inserted">amend your</carina-action-element><carina-action-element class="ng-star-inserted"> detailed Financial Disclosure statement. This is published with the article. It must therefore be completed in full sentences and contain the exact wording you wish to be published.</carina-action-element>

4) Figure 4D: Please confirm (a) that you are the photographer; or (b) provide written permission from the photographer to publish the photo(s) under our CC BY 4.0 license.

5) Figures 1A and 3A:  Please confirm whether you drew the images / clip-art within the figure panels by hand. If you did not draw the images, please provide (a) a link to the source of the images or icons and their license / terms of use; or (b) written permission from the copyright holder to publish the images or icons under our CC BY 4.0 license. Alternatively, you may replace the images with open source alternatives. See these open source resources you may use to replace images / clip-art:

- https://commons.wikimedia.org

- https://openclipart.org/

**Reviewers' Comments:**

Reviewer's Responses to Questions

**Part I - Summary**

Please use this section to discuss strengths/weaknesses of study, novelty/significance, general execution and scholarship.

Reviewer #1: In this short report, Frankel and colleagues employ a Citrobacter rodentium (CR) infection model in IL-22–knockout (Il22⁻/⁻) mice to investigate the contribution of immunopathology, especially neutrophil responses, to lethal infection outcomes. The study addresses an important question and provides valuable insights into the mechanisms underlying host-driven pathology during enteric infection.

The authors demonstrate that markedly increased neutrophil accumulation and activity in the colonic mucosa are associated with progressive colonic pathology during CR infection. Furthermore, their use of the ΔespO CR strain, lacking EspO (previously implicated in neutrophil recruitment), is another strength. The finding that ΔespO infection leads to substantially reduced neutrophil accumulation and decreased lethality provides evidence that EspO contributes to neutrophil-driven pathology rather than bacterial fitness. Overall, the data support the conclusion that excessive neutrophil responses are key drivers of lethality in Il22⁻/⁻ mice, and that EspO amplifies this pathology within a vulnerable epithelial environment.

The experiments are controlled, and the manuscript is clearly written and easy to follow. With a few revisions, the study would be further strengthened:

In summary, this is a well-executed and insightful study that will be of interest to researchers in mucosal immunology and host–pathogen interactions. Addressing the points above would further enhance the clarity and impact of the manuscript.

Reviewer #2: The study by Mishra et al. explores the role of neutrophils in lethality observed in Il22⁻/⁻ mice upon Citrobacter rodentium infection. The authors used espO mutant CR strain to demonstrate that higher mortality seen in CR infected Il22⁻/⁻ mice is due to excessive neutrophil accumulation. However, the study provides limited evidence to support that claim, lacks a clear mechanism, and leaves several issues unaddressed.

Reviewer #3: The short report from Mishra et al aims to elucidate the contribution of neutrophil responses to lethal C. rodentium infection in IL-22-/- mice. After showing that netrophil responses are increased, the authors use a C. rodentium mutant in the effector EspO to infect IL-22-/- mice. Infection with the espO mutant results in limited neutrophil accumulation to the mucosa, and lethality is rescued.

Overall, the study has the merit to continue elucidating the mechanism by which IL-22-/- mice succumb to C. rodentium infection, and is the logical continuation of prior work from this group. Using the espO mutant is a clever way to reduce neutrophil recruitment, even uncoupling it from other pathogen effects, for instance on tight junctions. The experiments are overall well conducted and interpreted.

I only have one issue with the interpretation of proteomics data, as discussed below.

**********

**Part II – Major Issues: Key Experiments Required for Acceptance**

Please use this section to detail the key new experiments or modifications of existing experiments that should be absolutely required to validate study conclusions.

Generally, there should be no more than 3 such required experiments or major modifications for a "Major Revision" recommendation. If more than 3 experiments are necessary to validate the study conclusions, then you are encouraged to recommend "Reject".

Reviewer #1: • Inclusion of an EspO chromosomally complemented CR strain in the background of ΔespO CR would provide important validation of the key findings presented in Figures 3 and 4.

Reviewer #2: The CR infection also appears to be associated with accumulation of other immune cells, including monocytes/macrophages, dendritic cells, and T cells. Did the authors assess differences in these populations between Il22⁻/⁻ and Il22⁺/⁺ mice? Analysis of these populations could provide additional insight. Could the authors clarify the rationale for focusing primarily on neutrophils, and whether the potential contributions of other immune cells were considered?

Previous work from the group suggests that CRΔespO reduces the expression of neutrophil-recruiting factors and neutrophil infiltration. In this study, Il22⁻/⁻ mice infected with CRΔespO show improved survival, despite no apparent change in epithelial damage. To better support a role for neutrophils in lethality, neutrophil depletion at early and later stages of CRwt infection could be informative.

Is this phenomenon independent of neutrophil development and maturation?

Can the bacteria still disseminate to other tissues?

Reviewer #3: The authors perform proteomics of colonic epithelial cells at day 6 post-infection. However, as shown in fig. 2, at this timepoint there is a significant recruitment of neutrophils, and it is thus possible that the "distinct epithelial proteomic signature in IL-22ko mice" actually includes neutrophil proteins. This is evident, for example, in Fig. 1G, where the "neutrophil granule/oxidative burst module" includes neutrophil proteins; for example, MPO is not an epithelial signature. Similarly, some of the acute phase/ S100 proteins are likely from neutrophils. I thus recommend that the authors rewrite this portion of the manuscript addressing the fact that the epithelial proteome likely includes neutrophil proteins; if not, additional controls to show the lack of neutrophil protein contamination need to be performed.

**********

**Part III – Minor Issues: Editorial and Data Presentation Modifications**

Please use this section for editorial suggestions as well as relatively minor modifications of existing data that would enhance clarity.

Reviewer #1: • The title could be refined to highlight “EspO-dependent dysregulation of neutrophil responses,” which would better reflect the central finding of the study.

• In the second paragraph of the Introduction (lines 56–62), it would be helpful to acknowledge the reported gut microbiota–independent lethality of CR infection in germ-free Il22⁻/⁻ mice (PMID: 39630719), to further contextualize the work.

Reviewer #2: The methods should be described in more detail rather than simply referring to “as previously described.”

Please correct the typos in Line # 84 (“of uninfected Il22⁺/⁺ and Il22⁻/⁻ mice”) and Line # 288 (“with 4 and 5 mice each per group”).

Could the authors provide higher-magnification overlays for Figures 2D, 3D, and 4B?

Figure 4 would benefit from inclusion of histological scoring.

Reviewer #3: (No Response)

**********

PLOS authors have the option to publish the peer review history of their article (what does this mean?). If published, this will include your full peer review and any attached files.

If you choose “no”, your identity will remain anonymous but your review may still be made public.

**Do you want your identity to be public for this peer review?**  For information about this choice, including consent withdrawal, please see our Privacy Policy.

Reviewer #1: No

Reviewer #2: No

Reviewer #3: No

[NOTE: If reviewer comments were submitted as an attachment file, they will be attached to this email and accessible via the submission site. Please log into your account, locate the manuscript record, and check for the action link "View Attachments". If this link does not appear, there are no attachment files.]

**Figure resubmission:**

**Reproducibility:**

To enhance the reproducibility of your results, we recommend that authors of applicable studies deposit laboratory protocols in protocols.io, where a protocol can be assigned its own identifier (DOI) such that it can be cited independently in the future. Additionally, PLOS ONE offers an option to publish peer-reviewed clinical study protocols. Read more information on sharing protocols at https://plos.org/protocols?utm_medium=editorial-email&utm_source=authorletters&utm_campaign=protocols

# Author comment: Author response to Decision Letter 1

**DOI:** 10.1371/journal.ppat.1014567.r002

15 Jul 2026

Attachment Submitted filename: PP-D-26-00394_Point to Point_Response.pdf

# Peer review: Decision Letter 1

**DOI:** 10.1371/journal.ppat.1014567.r003

**Authors:** Matthew C. Wolfgang, William Navarre

22 Aug 2026

Dear Dr Mishra,

We are pleased to inform that your manuscript, "EspO-mediated neutrophil accumulation drives lethality during Citrobacter rodentium infection in interleukin-22-deficient mice", has been editorially accepted for publication at PLOS Pathogens.

Before your manuscript can be formally accepted and sent to production, you will need to complete our formatting changes, which you will receive by email within a week. Please note that your manuscript will not be scheduled for publication until you have made the required changes.

IMPORTANT NOTES

(1) Copyediting and Proofreading: The corresponding author will receive a typeset proof for review, to ensure errors have not been introduced during production. Please review the PDF proof of your manuscript carefully, as this is the last chance to correct any errors. Please note that major changes, or those which affect the scientific understanding of the work, will likely cause delays to the publication date of your manuscript.

(2) Appropriate Figure Files: Please remove all name and figure # text from your figure files. Please also take this time to check that your figures are of high resolution, which will improve the readbility of your figures and help expedite your manuscript's publication. Please note that figures must have been originally created at 300dpi or higher. Do not manually increase the resolution of your files. For instructions on how to properly obtain high quality images, please review our Figure Guidelines, with examples at: http://journals.plos.org/plospathogens/s/figures.

We encourage you to use PACE (http://pace.apexcovantage.com/), a figure-checking tool which helps authors ensure all figures meet PLOS requirements so that figures can publish in the highest quality. PACE is a digital diagnostic and conversion tool for figure files. It will provide information about any failed check(s) and, if able, will automatically convert the figure into an acceptable file that passes quality checks.

To use PACE, you must first register as a user. Then, login and navigate to the UPLOAD tab, where you will find detailed instructions on how to use the tool.

(3) Striking Image: Please upload a striking still image to accompany your article if one is available (you can include a new image or an existing one from within your manuscript). Should your paper be accepted, this image will be considered for our monthly issue image and may also appear on our website to feature your article. Please upload this as a separate file, selecting "striking image" as the file type upon upload. Please also include a separate "Other" file with a caption, including credits and any potential copyright information. Please do not include the caption in the main article file. If your image is from someone other than yourself, please ensure that the artist has read and agreed to the terms and conditions of the Creative Commons Attribution License at http://journals.plos.org/plospathogens/s/content-license. Please note that PLOS cannot publish copyrighted images.

(4) Press Release or Related Media: If you or your institution will be preparing press materials for this manuscript, please inform our press team in advance at plospathogens@plos.org as soon as possible. We ask that you contact us within one week to plan ahead of our fast Production schedule. If you need to know your paper's publication date for related media purposes, you must coordinate with our press team, and your manuscript will remain under a strict press embargo until the publication date and time.

(5) Update your Profile Information: Now that your manuscript has been provisionally accepted, please log into Editorial Manager and update your profile, if needed. Go to http://www.editorialmanager.com/ppathogens, log in, and click on the "Update My Information" link at the top of the page. Please update your user information to ensure an efficient production and billing process.

(6) LaTeX users only: Our staff will ask you to upload a TEX file in addition to the PDF before the paper can be sent to typesetting, so please carefully review our Latex Guidelines http://journals.plos.org/plospathogens/s/latex in the meantime.

Thank you again for supporting Open Access publishing; we are looking forward to publishing your work in PLOS Pathogens.

Best regards,

William Navarre

Academic Editor

PLOS Pathogens

Matthew Wolfgang

Section Editor

PLOS Pathogens

Dominique Soldati-Favre

Editor-in-Chief

PLOS Pathogens

***********************************************************

Both reviewers were satisfied with the revisions.

Reviewer's Responses to Questions

**Part I - Summary**

Please use this section to discuss strengths/weaknesses of study, novelty/significance, general execution and scholarship.<br/><br/>

Reviewer #1: (No Response)

Reviewer #2: The authors have adequately addressed all of my comments. I have no further questions.

--------------------

**Part II – Major Issues: Key Experiments Required for Acceptance**

Please use this section to detail the key new experiments or modifications of existing experiments that should be absolutely required to validate study conclusions.<br/><br/>Generally, there should be no more than 3 such required experiments or major modifications for a "Major Revision" recommendation. If more than 3 experiments are necessary to validate the study conclusions, then you are encouraged to recommend "Reject".<br/><br/>

Reviewer #1: (No Response)

Reviewer #2: (No Response)

--------------------

**Part III – Minor Issues: Editorial and Data Presentation Modifications**

Please use this section for editorial suggestions as well as relatively minor modifications of existing data that would enhance clarity.<br/><br/>

Reviewer #1: (No Response)

Reviewer #2: (No Response)

--------------------

PLOS authors have the option to publish the peer review history of their article (what does this mean?). If published, this will include your full peer review and any attached files.

If you choose “no”, your identity will remain anonymous but your review may still be made public.

**Do you want your identity to be public for this peer review?**  For information about this choice, including consent withdrawal, please see our Privacy Policy.

Reviewer #1: No

Reviewer #2: No

# Editor report: Acceptance letter

**DOI:** 10.1371/journal.ppat.1014567.r004

**Authors:** Matthew C. Wolfgang, William Navarre

Dear Dr Mishra,

We are delighted to inform you that your manuscript, "EspO-mediated neutrophil accumulation drives lethality during Citrobacter rodentium infection in interleukin-22-deficient mice," has been formally accepted for publication in PLOS Pathogens.

We have now passed your article onto the PLOS Production Department who will complete the rest of the pre-publication process. All authors will receive a confirmation email upon publication.

Thank you again for supporting open-access publishing; we are looking forward to publishing your work in PLOS Pathogens.

Best regards,

Dominique Soldati-Favre

Editor-in-Chief

PLOS Pathogens
